# Effect of Light Quality on Metabolomic, Ionomic, and Transcriptomic Profiles in Tomato Fruit

**DOI:** 10.3390/ijms232113288

**Published:** 2022-10-31

**Authors:** Lingran Xiao, Tomoki Shibuya, Toshihiro Watanabe, Kazuhisa Kato, Yoshinori Kanayama

**Affiliations:** 1 Graduate School of Agricultural Science, Tohoku University, Aoba-ku, Sendai 980-8572, Japan; 2 Faulty of Agriculture, Yamagata University, Tsuruoka 997-8555, Japan; 3 Research Faculty of Agriculture, Hokkaido University, Sapporo 060-8589, Japan

**Keywords:** Micro-Tom, light quality, metabolome, ionome, transcriptome

## Abstract

Light quality affects plant growth and the functional component accumulation of fruits. However, there is little knowledge of the effects of light quality based on multiomics profiles. This study combined transcriptomic, ionomic, and metabolomic analyses to elucidate the effects of light quality on metabolism and gene expression in tomato fruit. Micro-Tom plants were grown under blue or red light-emitting diode light for 16 h daily after anthesis. White fluorescent light was used as a reference. The metabolite and element concentrations and the expression of genes markedly changed in response to blue and red light. Based on the metabolomic analysis, amino acid metabolism and secondary metabolite biosynthesis were active in blue light treatment. According to transcriptomic analysis, differentially expressed genes in blue and red light treatments were enriched in the pathways of secondary metabolite biosynthesis, carbon fixation, and glycine, serine, and threonine metabolism, supporting the results of the metabolomic analysis. Ionomic analysis indicated that the element levels in fruits were more susceptible to changes in light quality than in leaves. The concentration of some ions containing Fe in fruits increased under red light compared to under blue light. The altered expression level of genes encoding metal ion-binding proteins, metal tolerance proteins, and metal transporters in response to blue and red light in the transcriptomic analysis contributes to changes in the ionomic profiles of tomato fruit.

## 1. Introduction

Light is a vital environmental factor that greatly influences plant growth and development. As a characteristic of light, light quality, also called spectral composition, is effective for growth, morphogenesis, and other physiological processes in any plant’s development [1,2,3]. As a method of manipulating light quality, photo-selective netting or a film able to manipulate the spectral composition by adjusting the porosity and color of the nets [4] is applied to improve the yield and quality of horticultural plants. For example, red and yellow nets can enhance the yield of sweet pepper [5] and pearl and yellow net can help maintain the fruit quality of pepper during postharvest [6]. Another way to manipulate light quality is to use artificial light. Variable natural light has a great influence on plant production. Limited solar radiation usually occurs on cloudy days or at high latitudes in winter, restraining plant growth and development. Artificial light is applied to supplement radiation to enhance yield and ensure the stable production of leafy vegetables year-round [7]. In a controlled plant cultivation system, artificial light is indispensable for photoautotrophic plant growth like a plant factory [8]. As a rapidly developed lighting technology, light-emitting diodes (LEDs) with a long lifespan, little heat, environmentally friendly, and high-energy efficiency have become a good choice of artificial light in cultivation. Besides, LEDs can also generate narrow wavelengths of light, making it convenient to manipulate light quality to optimize plant quality and study the influence of light quality [9].

To make good use of light quality to manage plant production, it is necessary to have comprehensive knowledge of plant responses to various light qualities. Flowering is regulated by light quality; moreover, blue, red, and far-red wavelengths play crucial roles in plant photomorphogenesis and anthesis in *Arabidopsis* and horticultural plants [10]. Blue and far-red light promote flowering in *Arabidopsis* and *Eustoma* [11,12]. In addition to flowering, light quality can influence plant growth and metabolite accumulation in leafy vegetables [13]. The fresh and dry weight of lettuce plants treated with red light is reduced compared to plants treated with blue light [14]. Changes in red/blue ratios influence the photosynthetic rate, fresh weight, and total phenol concentration in lettuce plants [15]. In addition to leafy vegetables, the effect of light quality on fruit vegetables is of high concern. Tomato (*Solanum lycopersicum*) is the most produced vegetable in the world and a model plant for research on fleshy fruits. Tomato fruits contain various health-promoting components, including carotenoids, vitamins, polyphenols, flavonoids, and potassium [16]. Most reports have concentrated on the influence of light quality on plant morphology or the postharvest quality of fruits [17,18,19,20,21]. However, little information on the effects of light quality on tomatoes based on multiomics studies, including metabolomics, ionomics, and transcriptomics during fruit development, can be found.

High-throughput methods, metabolomic analysis, ionomic analysis, and RNA-sequencing (RNA-seq) provide huge data sets to monitor the accumulation and fluctuation of metabolites, minerals, and nutrients and gene expression [22,23]. The metabolites of tomato fruit have been studied in transgenic plants, cultivated varieties, and wild species [24,25,26]. Metabolomic analysis has been used to identify metabolites in wild species potentially important in reference to stress responses and nutritional value [23,26]. A coexpression analysis by RNA-seq indicated expression patterns of several transcription factors relevant to genes involved in the biosynthesis of ascorbic acid, carotenoids, and flavonoids [27]. Ionomic analysis indicated the similarity in element concentrations across species by classifying the element profiles in leaves and other organs [28]. In the past years, most studies have only used one high-throughput method to analyze horticultural crops. With respect to the effects of light quality, little information can be found based on omics analyses. However, a multiomics study of transcriptomic and metabolomic or ionomic data can facilitate the understanding of metabolite and element networks and promote the identification of genes controlling the metabolism or element absorption and translocation [29,30]. Therefore, in this study, to elucidate the effects of light quality on fruit metabolites and elements and the regulation mechanism, transcriptomic analysis was combined with ionomic and metabolomic analyses to determine the changes in metabolism and gene expression in response to red or blue light in tomato fruit. Moreover, to the authors’ best knowledge, there is no information about ionomic profiling response to light quality in tomatoes. Therefore, this study will provide fundamental knowledge of this domain.

Micro-Tom plants were grown under white fluorescent light during the vegetative growth period because tomato plants are unable to grow normally when irradiated with monochromatic light [31,32]. Monochromatic light, including blue or red LED, was used as a light source after anthesis for comparison. Meanwhile, white fluorescent light was also used for reference. Metabolomic, ionomic, and transcriptomic analyses were performed to determine their effects.

## 2. Results

### 2.1. Metabolomic Analysis

In this study, there were no significant differences in plant growth parameters, such as shoot dry weight and fruit yield, among the three light treatments (data not shown). Therefore, plants were grown under their respective PPFDs and this study aimed to focus on the quality of tomato fruit and investigate the effects of light quality on fruit metabolites and elements.

To investigate the effects of different light treatments on the metabolism of tomato fruit, the metabolites were identified and quantified based on the peak information, *m/z*, migration time, and retention time obtained from capillary electrophoresis-time-of-flight mass spectrometry (CE-TOFMS) and liquid chromatography time-of-flight mass spectrometry (LC-TOFMS). A total of 296 peaks (CE-TOFMS: 167, 82 cations, and 85 anions; LC-TOFMS: 129, 60 positives, and 69 negatives) were annotated using the human metabolome technologies (HMT) metabolite library and known-unknown library.

**Figure 1:**
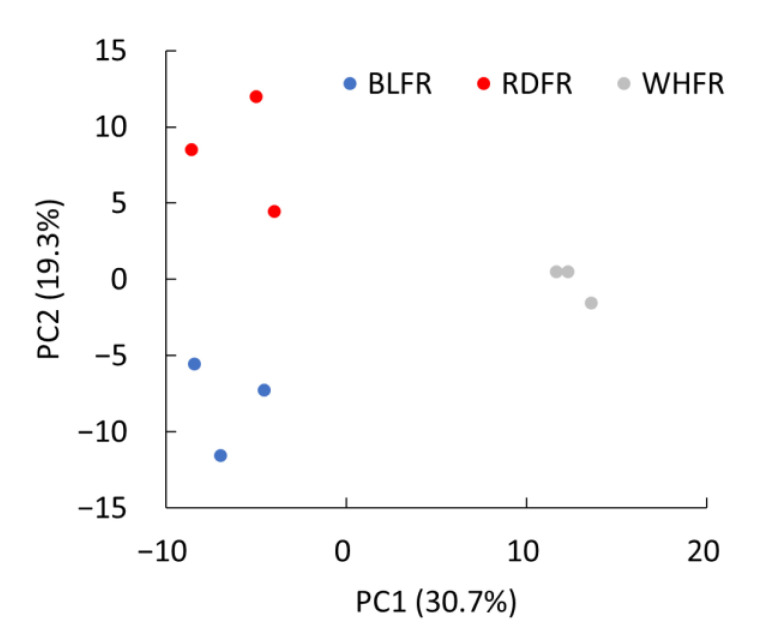
PCA of metabolic profiles in fruits with different light treatments. PCA scores are presented based on a combination of the first (PC1) and second (PC2) principal components and variances of each component in the sample set. BLFR, RDFR, and WHFR refer to blue, red, and white light-treated fruits.

**Figure 2:**
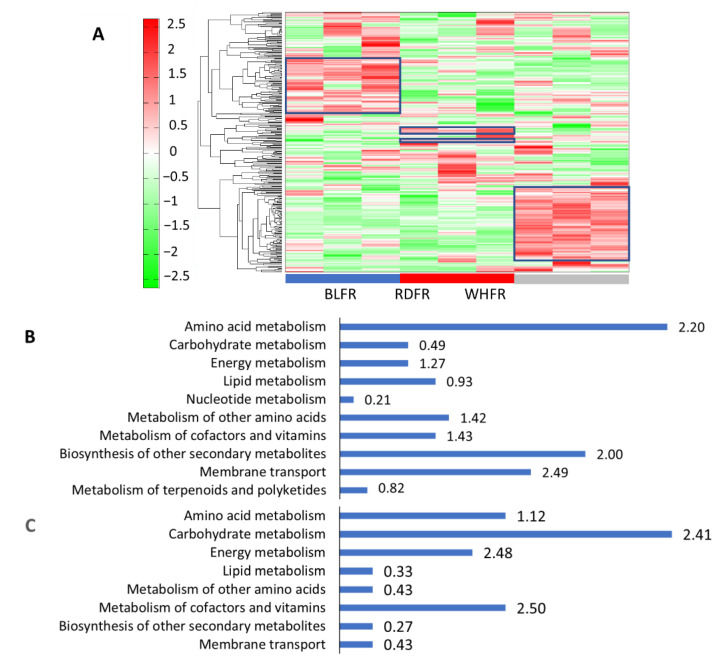

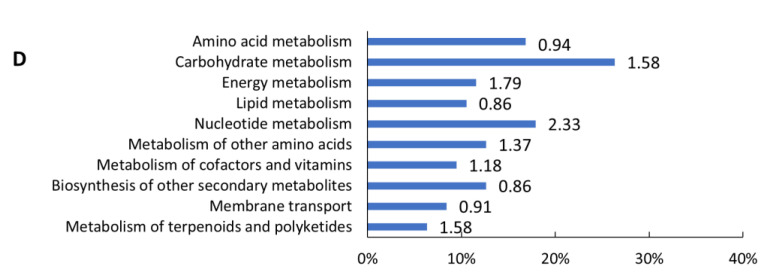
Metabolite profiles of fruits with different light treatments (**A**). In the heat map, red and green rectangles indicate higher and lower metabolite levels, respectively, than the mean of all metabolites. Values indicate the z-score in the legend on the left. Marked changes in the groups of metabolites due to a higher amount of metabolites in each light treatment were indicated by blue-boxed metabolite sets. BLFR, RDFR, and WHFR refer to blue, red, and white light-treated fruits, respectively. Functional classification of metabolites showed marked changes (blue-boxed) among the three light treatments. The bars show the percentage of metabolites classified into each functional category in total metabolites in the blue (**B**), red (**C**), and white (**D**) light-treated groups. Values beside the bars in each functional category indicate the enrichment value, which is the percentage ratio in each category in this figure to that in all detected metabolites.

Principal component analysis (PCA) and hierarchical clustering analysis (HCA) were performed on the 296 annotated metabolites to facilitate comparing tomato fruit among the three light treatments. The PCA result revealed that the first principal component (PC1; 30.7%) showed high similarity between fruits treated with blue light (BLFR) and red light (RDFR), whereas the second principal component (PC2; 19.3%) showed a marked difference between BLFR and RDFR, and PC2 scores in fruits treated with white light (WHFR) were between BLFR and RDFR (Figure 1). Therefore, it can be concluded that the fruit metabolite concentration was influenced by the difference between the two monochromatic lights and by the difference between monochromatic and mixed light. The HCA result was presented in a heatmap to identify metabolic profiles in fruits treated with different light treatments (Figure 2A). A dendrogram was produced based on similarity, and the metabolites were divided into some groups. The groups of metabolites with apparent differences in color on the heat map, which are indicated as the blue-boxed metabolite sets in Figure 2A, showed different patterns due to a higher amount of metabolites in each light treatment. Metabolites of the groups were mapped to tomato metabolic pathways in the Kyoto Encyclopedia of Genes and Genomes (KEGG), and the proportions of metabolites in each pathway are described in Figure 2B–D. In the circled group of blue light treatment, most metabolites were involved in amino acid metabolism (39.3%), followed by secondary metabolite biosynthesis (29.5%). In the circled group of red and white light treatment, most metabolites were involved in carbohydrate metabolism, and the proportions were 40.0% and 26.3%, respectively (Figure 2). The enrichment value of metabolites in groups of blue light treatment in amino acid metabolism and secondary metabolite biosynthesis was also high. Besides, the highest enrichment value was observed at membrane transport (2.49). The value of carbohydrate metabolism was obviously high for the red and white light-treated groups. In contrast, the enrichment of the blue light-treated group in carbohydrate metabolism was low (0.49). Higher enrichment values were observed in energy metabolism (2.48) and metabolism of cofactors and vitamins (2.50) in the red light-treated group.

**Figure 3:**
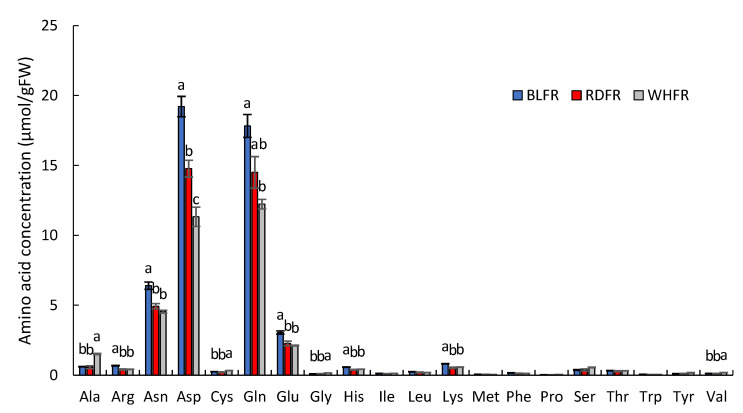
Amino acid concentration in fruits with different light treatments. Values indicate the mean ± standard error (SE; *n* = 3). Different letters indicate significant differences at *p* < 0.05 by Tukey’s test. BLFR, RDFR, and WHFR refer to blue, red, and white light-treated fruits, respectively.

**Figure 4:**
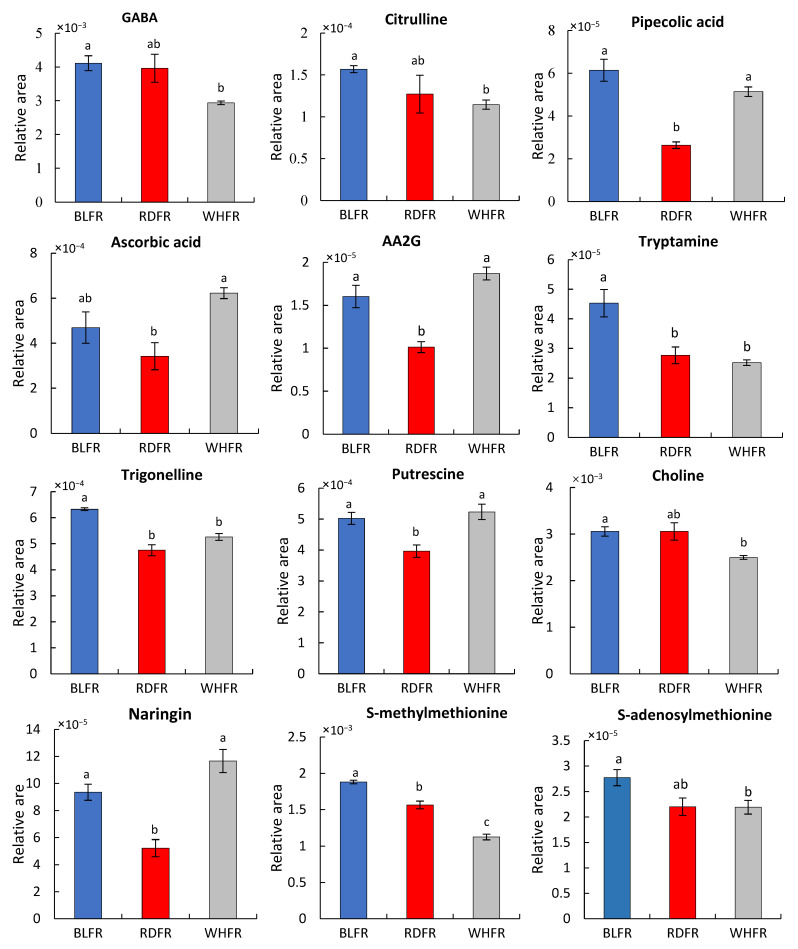
GABA, citrulline, pipecolic acid, ascorbic acid, AA2G, tryptamine, trigonelline, putrescine, choline, naringin, S-methylmethionine, and S-adenosylmethionine concentration in fruits treated with different lights. Values indicate the mean ± SE (*n* = 3). Different letters indicate significant differences at *p* < 0.05 by Welch’s *t*-test. BLFR, RDFR, and WHFR refer to blue, red, and white light-treated fruits, respectively.

Amino acids are essential food compounds. In addition to general nutritional value, they also provide several health benefits, such as antimutagenicity [33] and reduction in blood sugar [34]. In Figure 3, the concentration of six proteinogenic amino acids, including aspartate (Asp) and glutamate (Glu), in BLFR was significantly higher than in RDFR. In contrast, there were four amino acids with higher concentrations in WHFR. Two nonprotein amino acids, γ-aminobutyric acid (GABA) and citrulline, displayed higher concentrations in BLFR than WHFR (Figure 4). The cyclic amino acid pipecolic acid is a nonproteogenic amino acid generated from lysine (Lys), which showed higher concentrations in BLFR than RDFR (Figure 4).

In this study, the accumulation of two vitamins, ascorbic acid and tocopherol, in fruits was also influenced by light quality. Tocopherol concentration improved in BLFR. A-Tocopherol showed higher levels in BLFR than RDFR, whereas γ-tocopherol showed higher levels in BLFR than WHFR (see Discussion). However, ascorbic acid showed lower levels in RDFR than WHFR (Figure 4). Consequently, blue light plays an important role in tocopherol biosynthesis, whereas red light suppresses ascorbic acid accumulation.

**Figure 5:**
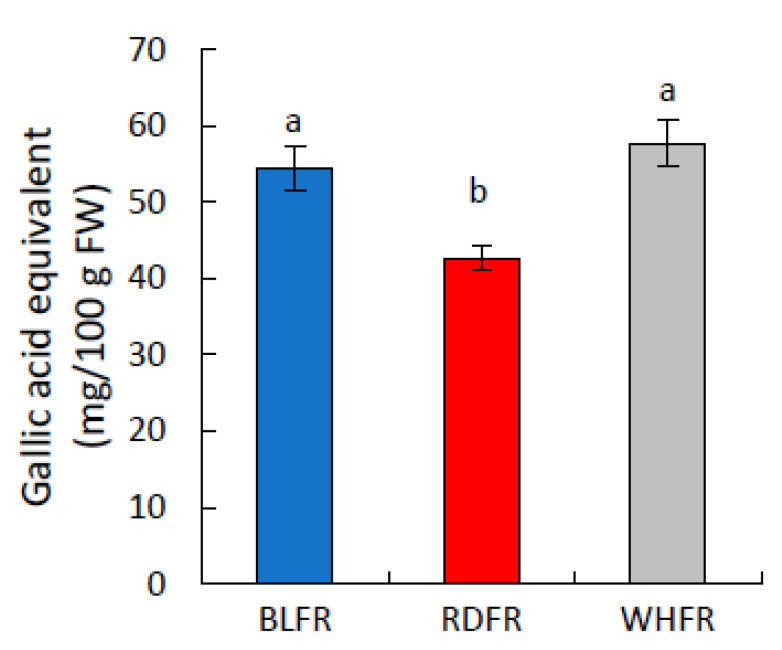
Influence of light quality on the total phenol concentration in fruits. Results are the mean ± SE (*n* = 3). Different letters indicate significant differences at *p* < 0.05 by Tukey-Kramer’s test. BLFR, RDFR, and WHFR refer to blue, red, and white light-treated fruits, respectively.

The biosynthesis of the other secondary metabolites was active under blue light. This study focused on high accumulated metabolites in this treatment. Ascorbic acid 2-glucoside (AA2G) exhibited markedly more elevated levels in BLFR than RDFR (Figure 4). Trigonelline and tryptamine accumulation is significantly higher in BLFR than in WHFR or RDFR in tomato fruit (Figure 4). Putrescine levels were relatively stimulated in BLFR and WHFR (Figure 4), indicating that blue light is crucial in putrescine accumulation. Choline levels were markedly higher in BLFR than in WHFR (Figure 4). Naringin, a citrus flavonoid, is detected in tomato fruit and displayed higher concentrations in BLFR than RDFR (Figure 4). The total phenolic compound concentration was stimulated in tomato fruit in BLFR than RDFR (Figure 5). S-methylmethionine and S-adenosylmethionine had higher accumulation in BLFR than WHFR, and S-methylmethionine accumulation was also higher in BLFR than RDFR (Figure 4).

### 2.2. Ionomic Analysis

**Figure 6:**
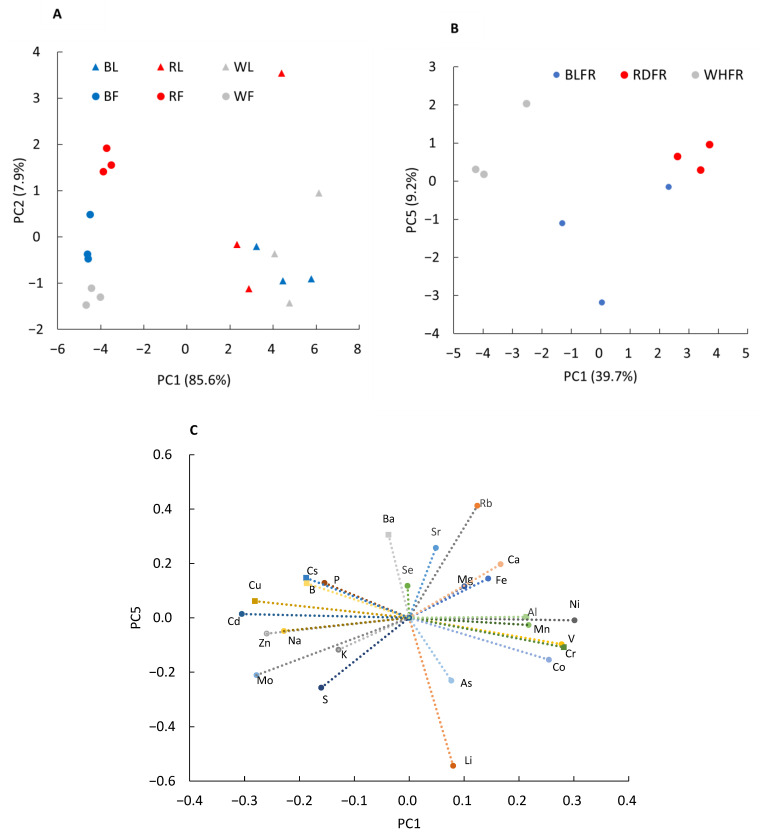
PCA of element concentration in leaves and fruits (**A**). BL and BF, blue-light-treated leaves and fruits; RL and RF, red light-treated leaves and fruits; WL and WF, white light-treated leaves and fruits. PCA (**B**) and factor loading (**C**) of element concentration in fruits. BLFR, RDFR, and WHFR refer to blue, red, and white light-treated fruits, respectively.

Uptake and translocation of mineral nutrients play a vital role in plant growth and human health [35]. Therefore, the accumulation of elements in tomato leaves and fruits was analyzed under different light qualities. PCA was performed using the concentration of 25 elements. The total contribution rate of PC1 and PC2 was 94%. PC1 showed a marked difference between leaves and fruit, whereas PC2 showed the difference among different light qualities (Figure 6A). When all data were used, the contribution of PC1, which indicates the difference between leaves and fruits, was too large to make it difficult to examine the effects of light quality. Therefore, PCA was conducted again using only data from fruits. A scatter plot was created using PC1 and the fifth principal component (PC5) as shown in Figure 6B, whose values were separated in the treatment interval. According to the result, PC1, which explained 39.7% of the total variance, showed a marked difference among light treatments in fruit. The scatter plot was also created using the factor loadings of each element using PC1 and PC5 as shown in Figure 6C, indicating that many elements were affected by light quality.

**Figure 7:**
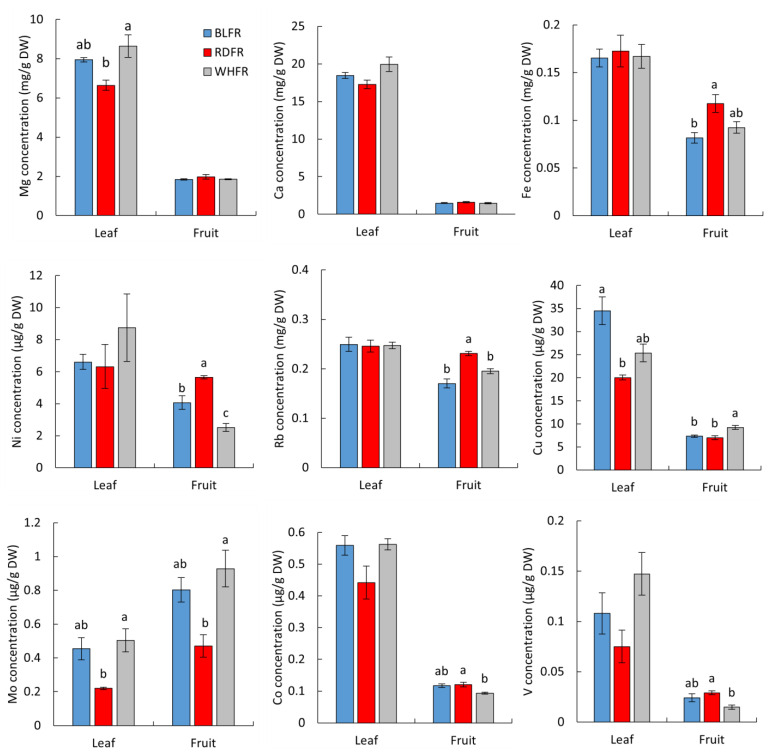
Mg, Ca, Fe, Ni, Rb, Cu, Mo, Co, and V concentrations of leaves and fruits treated with different lights. Values indicate the mean ± SE (*n* = 3). Different letters indicate significant differences at *p* < 0.05 by Tukey-Kramer’s test. BLFR, RDFR, and WHFR refer to blue, red, and white light treatments, respectively.

Among the macroelements, the Mg concentration in leaves was significantly lower in RDFR than in WHFR. The Ca concentration in leaves, which is also a group 2 element, showed a similar trend to that of Mg, whereas there was no significant difference between the treatments (Figure 7). In fruit, no significant difference can be found in the microelement concentration among different light treatments (Figure 7, data not shown for P, K, and S). With regard to microelements, the Fe, Ni, and Rb concentration in fruits in RDFR was markedly higher than that of BLFR (Figure 7). The Cu concentration showed a reverse pattern in three light treatments between leaves and fruits. In BLFR, the Cu concentration was higher in leaves and lower in fruits. The ratio of fruits to leaves was also lower in BLFR than in other treatments (Figure S2). The Ni concentration in fruits was higher in the order of RDFR, BLFR, and WHFR, and the ratio of fruits to leaves varied among the treatments (Figure S2). The Cu and Ni results suggested that light quality may affect the retranslocation of these elements. In contrast, the Mo concentration in both leaves and fruits was lower in RDFR than in WHFR (Figure 7). Co and V significantly accumulated in fruits in RDFR than in WHFR (Figure 7). Mo was the only element in which the ratio of fruits to leaves was a positive value (Figure S2), whereas eggplant, which belongs to the same family of tomato, showed a negative ratio of fruits to leaves in the Mo concentration [28].

### 2.3. Transcriptomic Analysis

Tomato whole-transcriptome sequencing was performed on all nine paired-end samples. After quality control, trimmed reads mapping, transcript assembly, and expression profiling, genes expressed differentially were filtered using statistical hypothesis testing on three comparisons. Data quality check excluded 13,974 genes from a total of 31,366 genes, and only 17,392 genes were used for analysis. Statistical analysis was performed with an independent *t*-test (*p* < 0.05) per comparison pair, and relevant genes were considered.

**Figure 8:**
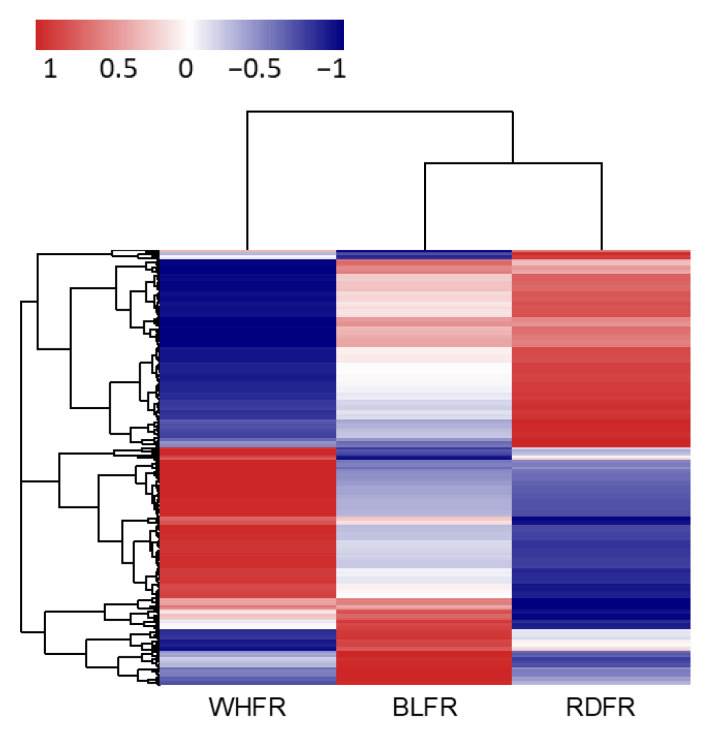
Differentially expressed genes of fruit with different light treatments. Red and blue rectangles indicate higher and lower levels, respectively, than the mean of all treatments. Values indicate the z-score in the legend. BLFR, RDFR, and WHFR refer to blue, red, and white light-treated fruits, respectively.

The significant results with |fold change (FC)| ≥ 2 were selected and used for hierarchical clustering, volcano plots, and enrichment analyses. In total, 95, 170, and 379 genes were selected in BLFR vs. RDFR, BLFR vs. WHFR, and RDFR vs. WHFR, respectively. For significant lists, HCA was performed to group similar samples and genes. These results were graphically depicted using heatmaps and dendrograms. The heat map indicated significantly different patterns in gene expression among the three treatments (Figure 8). Log_2_ FC and *p*-value were obtained from the comparison between two groups, such as BLFR and RDFR, plotted as volcano plot (Figure S3). In BLFR vs. RDFR, upregulated genes were more than downregulated genes, and a similar pattern could also be found in BLFR vs. WHFR. These results indicated that the expression of more genes was improved in blue light treatment. A microarray study in *Arabidopsis* exhibited similar results that changes in expression of ~20% transcriptional factors were found in response to blue light; moreover, upregulated genes were more than downregulated genes [36].

To identify the functions of these differentially expressed genes, gene-set enrichment analysis (GSEA) was performed based on Gene Ontology (GO), KEGG, and other functional annotation databases for 499 significant transcripts. This study focused on influences on fruit quality, so significant enrichment analysis results associated with the accumulation of quality-related metabolites and elements in various databases were picked up (Table 1). Genes involved in the biosynthesis of secondary metabolites were highly enriched in BLFR vs. RDFR (Table 1), indicating that secondary metabolite accumulation may be affected by light quality. Besides, differentially expressed genes were enriched in glycine, serine, and threonine metabolism, which belong to amino acid metabolism. In BLFR vs. WHFR, differential expression genes were markedly enriched in the amino sugar metabolic process, fructose metabolic process, and sucrose biosynthetic process (Table 1), indicating that carbohydrate metabolism was influenced by blue light treatment. This result was consistent with the result that carbohydrate metabolism was active under white light, as described in the metabolomic analysis, compared to blue light treatment. Besides, differentially expressed genes between BLFR and WHFR were markedly enriched in metal ion binding (Table 1). Between RDFR and WHFR, genes involved in fruit ripening were enriched, according to the GO enrichment results, suggesting that other wavelengths, except red light, included in white light play a crucial role in fruit ripening (Table 1). Since it has been suggested that ripening related to fruit quality is influenced by light quality and photoreceptors [32], the physiological mechanism are expected to be elucidated in the future.

## 3. Discussion

### 3.1. Amino Acid Metabolism

Red light influences the fruit color [37] and functional component accumulation in tomato fruit [19]. In this study, HCA showed that the metabolite levels in tomato fruit were greatly regulated by blue and red light (Figure 2), and the groups of metabolites with high levels were classified at three treatments. Amino acid metabolism was promoted in blue light treatment. Furthermore, GSEA showed that differentially expressed genes between BLFR and RDFR were enriched in metabolic pathways for amino acids (Table 1).

Amino acids play several vital roles in plants other than protein biosynthesis. They are tightly connected with several other biosynthesis pathways and play significant roles during signaling processes and in plant stress response [38]. In tomato fruit, free amino acid content increases during the ripening process [39]. In this study, many amino acid concentrations of ripe fruits markedly increased under blue light according to metabolomic analysis, including proteinogenic and nonproteinogenic amino acids (Figure 3 and Figure 4). Blue light positively affected citrulline; that is, its metabolite concentration was highest in BLFR. Citrulline functions as a potent hydroxyl radical scavenger and an emerging biomarker in intestinal pathology and early diagnosis of rheumatoid arthritis [40,41,42]. Pipecolic acid functions in many aspects in microorganisms, plants, and animals, including the interactions between organisms, acting as a strong osmoprotectant, and responding to Ca deficiency when the proline level is low [43,44]. Moreover, GABA and arginine (Arg) also function as stress-related metabolites [45,46]. Glutamic acid (Glu) provides the characteristic “umami taste” to food [47]. Consequently, blue light affects the accumulation of various amino acids, influencing the taste of fruit and nutritional value of fruit quality and improving tomato resistance to environmental stress.

**Figure 9:**
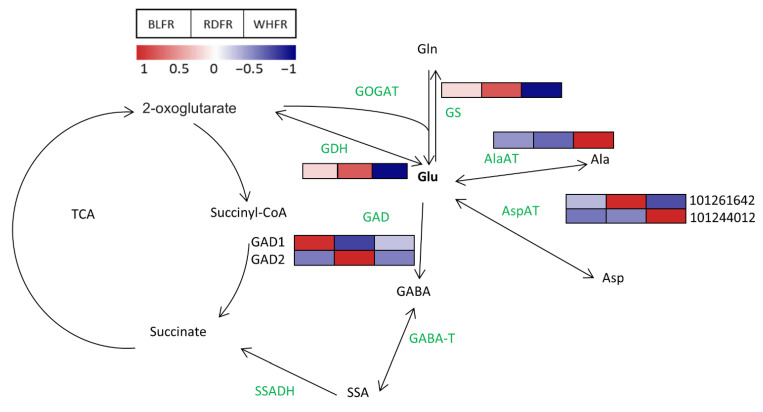
Transcriptomic profiles of genes involved in the Glu metabolism pathway. Red and blue rectangles indicate higher and lower levels, respectively, than the mean of all treatments. Values indicate the z-score in the legend. The experimental data were subjected to an independent *t*-test at *p* < 0.05. Significant differences are found in the transcription level of *GDH* in RDFR vs. WHFR, *GAD1* in BLFR vs. others, *GAD2* in RDFR vs. others, *AspAT* (Gene ID: 101261642) in RDFR vs. WHFR, *AspAT* (Gene ID: 101244012) in WHFR vs. others, *AlaAT* in RDFR vs. WHFR, and *GS* in RDFR vs. WHFR. BLFR, RDFR, and WHFR refer to blue, red, and white light-treated fruits, respectively.

Asp and Glu are quantitatively important amino acids in tomato fruit [48,49], and Glu is in the central position of amino acid metabolism in plants; that is, the α-amino group of Glu is transferred to other amino acids by a wide range of aminotransferases [50]. Accordingly, this study focused on Glu and attempted to determine the mechanism of its high accumulation in blue light treatment at the transcript level. In Ala, Asp, and Glu metabolism, there were nine metabolites and eight enzymes mapped to the pathway from the KEGG database (Figure 9). In addition to Glu, Asp, asparagine (Asn), Arg, histidine (His), and Lys were significantly higher in BLFR compared to RDFR (Figure 3). In regard to genes related to Glu metabolism, inorganic nitrogen was assimilated by glutamine synthetase/Glu synthase (GS/GOGAT) circle and incorporated into Ala, Asp, GABA, and other amino acids [51]. In plants, most Glu is synthesized by the GS/GOGAT circle [52]. The *NADH-GOGAT* transcript level in BLFR was 1.94-fold of that in RDFR, although the difference was insignificant (data not shown). Because the high activity of GOGAT in blue light-treated pakchoi supports this study [53], further research is required to confirm this using more samples. Glutamate decarboxylase (GAD) genes encode the GAD that catalyzes the synthesis of GABA and carbon dioxide (CO_2_) from Glu, which is an irreversible reaction [54]. Three genes (*GAD1, GAD2,* and *GAD3*) identified in tomatoes are likely to become the key genes in GABA biosynthesis, in which GAD2 and GAD3 appear to be major isoforms in regulating GABA levels [54]. As the substrate of this reaction, the Glu level can also be regulated by GAD2 and GAD3. A previous study demonstrated that the introduction of *GAD* in antisense orientation in tomatoes improved the Glu level of nontransgenic plants [55]. The *GAD2* expression level was 1.88-fold downregulated and significantly lower in BLFR (Figure 9) compared to RDFR, which is supposed to contribute to high Glu concentration in BLFR.

### 3.2. Secondary Metabolism

Secondary metabolite biosynthesis was active in BLFR according to the functional classification of metabolites. The accumulation of many secondary metabolites greatly improved in BLFR, such as AA2G, choline, tryptamine, trigonelline, putrescine, S-adenosylmethionine, and S-methylmethionine (Figure 4). AA2G is a highly stable form of ascorbic acid, which can express ascorbic acid activity when hydrolyzed in vivo. Furthermore, it has an apoptotic ability to protect gastric epithelial cells from *Helicobacter pylori* infection [56,57]. Choline is an essential nutrient for the nervous system and membrane formation [58]. Putrescine is one of the main polyamines that play an important role in regulating plant growth and development and functions in biotic and abiotic stress responses [59]. Furthermore, this metabolite is involved in membrane transport [60]. The putrescine level was relatively stimulated in BLFR (Figure 4) and higher in WHFR than in RDFR, indicating that blue light is crucial in accumulating this metabolite. Genes related to putrescine biosynthesis were analyzed to figure out the mechanism. Putrescine is known to be synthesized in three routes, which include five enzymes in total, to catalyze the reactions [61]. Seven genes encoding these enzymes were detected, among which Arg decarboxylase gene *ADC1* (Gene ID: 543807) showed a 1.46-fold higher expression level in BLFR than RDFR (data not shown). *ADC1* can be a key gene regulating putrescine biosynthesis under blue light, although confirmation is necessary because of no significant difference in expression levels between light qualities. Tryptamine is a precursor of serotonin, a functional ingredient that plays a role as a neuromodulator or neurotransmitter and has antiobesity effects [23,62,63]. S-methylmethionine is a derivative of methionine with a positive effect on the development of resistance to abiotic and biotic stress [64,65,66] and plays a major role in phloem sulfur transport in plants [67]. S-adenosylmethionine is an essential cofactor that offers a methyl group in methylation reactions and is the second most widely used enzyme substrate after ATP [68,69]. Trigonelline plays a role in the regeneration of the neuronal network and memory improvement [70]. An earlier study reported that antioxidants, total phenolic compounds, and total flavonoids were high in harvested fruit and seedlings in tomato owing to the use of blue light [71,72]. The total phenolic compound concentration was higher in tomato fruit in BLFR than in RDFR (Figure 5), consistent with seedlings in the previous study. Naringin exhibits strong anti-inflammatory and antioxidant activities [73]. Consequently, blue light can enhance resistance to abiotic and biotic stress and improve fruit quality related to tomato-added value by increasing the accumulation of these functional secondary metabolites.

With regard to gene expression, 11 differently expressed genes were enriched in the biosynthesis of secondary metabolites in BLFR vs. RDFR. More than twofold differences in the expression levels were detected between the two light qualities (Table 1 and Table S1). Among them, the products of serine hydroxymethyltransferase 4 (SHMT) and glycerate dehydrogenase reactions are utilized in many crucial biosynthetic pathways, such as photorespiratory cycle, carbohydrate, and amino acid metabolism [74,75,76]. SHMT catalyzes the reversible exchange between serine and glycine, and a mitochondrial SHMT gene mutation in rice results in the blockage of the photorespiration pathway, thereby affecting the Calvin cycle and the efficiency of light energy [77]. These could be related to active carbon fixation under blue light (Table 1). SHMT is also important for generating one-carbon fragments for synthesizing nucleotides, methionine, thymidylate, choline, etc. [78]. Glycerate dehydrogenase is involved in the one-carbon metabolism as well. Glycerate dehydrogenase is a peroxisomal enzyme that catalyzes the NADH-dependent reduction of hydroxypyruvate into serine in the nonphosphorylated (glycerate) serine pathway [79]. The two differentially expressed genes are involved in glycine, serine, and threonine metabolism, which was active in BLFR (Table 1).

### 3.3. Calvin Cycle

**Figure 10:**
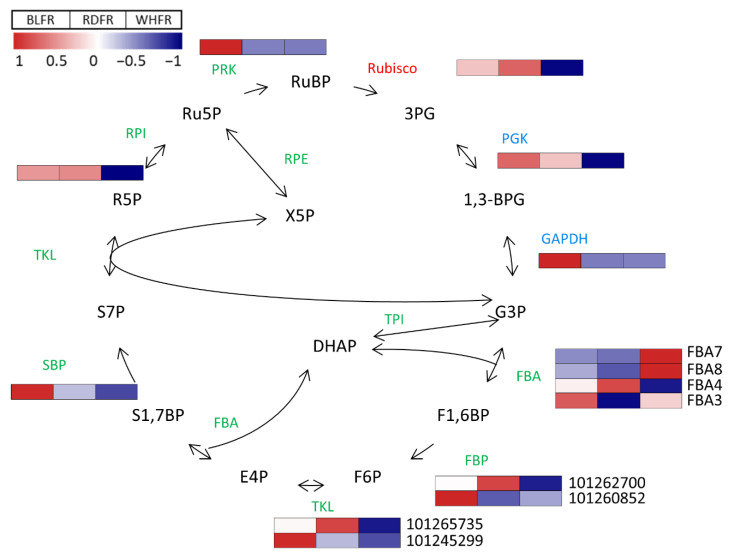

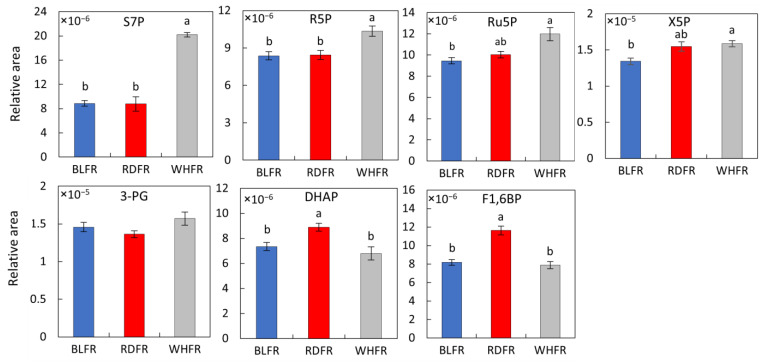
Transcriptomic profiles of carbon fixation (red), reduction (blue), and regeneration phase (green) and metabolic profiles of the Calvin cycle. Significant differences and >2 FC were found in the transcription level of *PRK*, *FBP* (Gene ID: 101260852), *FBA3*, and *GAPDH* in BLFR vs. RDFR. Significant differences and >2 FC were found in the transcription level of *PRK*, *FBP* (Gene ID: 101260852), *GAPDH*, *SBP*, and *TKL* (Gene ID: 101245299) in BLFR vs. WHFR. Significant differences and >2 FC were found in the transcription level of *rbcL* in RDFR vs. WHFR. Red and blue rectangles indicate higher and lower levels, respectively, than the mean of all treatments. Values indicate the z-score in the legend. Sedoheptulose 7-phosphate (S7P), ribose 5-phosphate (R5P), ribulose 5-phosphate (Ru5P), xylulose 5-phosphate (X5P), 3-phosphoglyceric acid (3-PG), dihydroxyacetone phosphate (DHAP), and fructose 1,6-diphosphate (F1,6BP) concentration in fruits treated with different light treatments. Values indicate the mean ± SE (*n* = 3). Different letters indicate significant differences at *p* < 0.05 by an independent *t*-test. BLFR, RDFR, and WHFR refer to blue, red, and white light-treated fruits, respectively.

Differentially expressed genes in BLFR vs. RDFR were found enriched in carbon fixation (Table 1), and most genes were involved in the Calvin cycle (Figure 10), a critical pathway in the whole plant metabolism, by which CO_2_ is incorporated into carbohydrates. In contrast to the gene expression pattern, the metabolite levels were lower or similar in BLFR compared to RDFR. DHAP and F1,6BP showed higher concentrations in RDFR compared to BLFR (Figure 10). Both metabolites play vital roles in energy metabolism (carbon fixation) and carbohydrate metabolism and participate in reactions catalyzed by fructose-1,6-bisphosphate aldolase (FBA). FBA plays a vital role in carbohydrate metabolism and energy metabolism, as it is an essential enzyme involved in glycolysis and gluconeogenesis pathways in the cytoplasm and the Calvin cycle in the plastid [80,81]. Light quality affects plant photosynthesis in many aspects. For example, blue light-treated plants increased stomatal opening, chlorophyll a/b ratios, photosynthetic electron-transport activity, and Rubisco activity [82,83]. The effects of light quality on plant photosynthesis are realized via alternation in the activity of the photosynthetic apparatus and fluctuation in the expression or activity of the Calvin cycle enzymes [84]. The tomato FBA family is found to eight members [85]. Five genes were detected, and only *FBA3*, which is localized in the chloroplast, was identified as differentially expressed gene between BLFR and RDFR (Figure 10). FBA is involved in response to environmental changes, such as drought, chilling, and light intensity [86,87,88]. Results indicated that the *FBA* expression levels also fluctuate in response to light quality, thereby influencing the Calvin cycle. Fructose 1,6-bisphosphatase (FBP) catalyzes the conversion of F1,6BP to F6P. Like FBA, FBP has two isoforms in the plastid and cytoplasm. The expression level enhanced in BLFR was the gene of FBP (Gene ID: 101260852) in the chloroplast (Figure 10) and correlated with lower F1,6BP levels. In addition, blue light increased the mRNA level of genes encoding other key enzymes, such as SBP, GAPDH, and PRK. Results were consistent; monochromatic blue light elevated Rubisco activities and transcriptional levels of several genes involved in the Calvin cycle compared to white light in cucumber leaves [84]. As metabolites in the Calvin cycle, DHAP and F1,6P concentrations decreased in BLFR. Low levels of metabolites were attributed to the high expression of genes (*FBA3, FBP*) involved in DHAP and F1,6P-related reactions. The chloroplastic FBP functions as a regulatory enzyme in the Calvin cycle because the low level of FBP activity led to a reduced growth rate and decreased yield [89]. A reduction of FBP activity improves the sucrose/starch ratio and leaf fresh weight in *Arabidopsis* and reduces the weight of ripe tomato fruit [90,91]. Although there was no significant difference in fruit weight in the three light treatments (data not shown), tomatoes altered the expression of genes encoding key enzymes in the Calvin cycle in response to blue light treatment, which may affect photosynthesis and other metabolisms. Metabolites and enzymes in the Calvin cycle are also involved in the pentose phosphate cycle and are closely related to other pathways of primary metabolism, such as glycolysis. Therefore, those metabolites and enzymes do not necessarily refer only to carbon dioxide fixation by photosynthesis and the results may suggest a new regulatory mechanism of carbohydrate metabolism via blue light.

### 3.4. Biosynthesis of Vitamins

**Figure 11:**
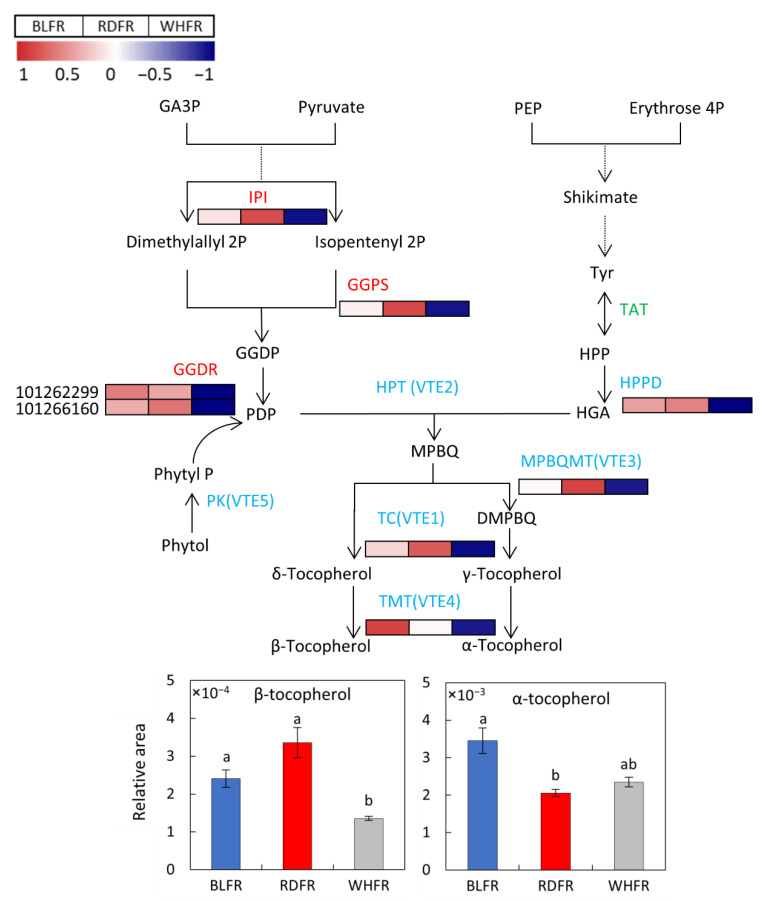
Transcriptomic profile and metabolic profiles of the tocopherol biosynthetic pathway, including methyl erythritol (MEP) pathway (red), Shikimate (SK) pathway (green), and vitamin E core pathway (blue). Significant differences were found in the transcription level of *IPI*, *GGPS*, *HPPD*, and *VTE3* in RDFR vs. WHFR, *VTE4* in BLFR vs. WHFR, and *VTE1, GGDR* (101262299; >2 FC) and *GGDR* (101266160) in WHFR vs. other treatments. Red and blue rectangles indicate higher and lower levels, respectively, than the mean of all treatments. Values indicate the z-score in the legend. β-tocopherol (B) and α-tocopherol (C) concentrations in fruits treated with different light treatments. Values indicate the mean ± SE (*n* = 3). Different letters indicate significant differences at *p* < 0.05 by an independent *t*-test. BLFR, RDFR, and WHFR refer to blue, red, and white light-treated fruits, respectively.

Ascorbic acid and tocopherol are two vitamins that catch consumers’ attention for their functional role as antioxidants, so there is great interest in developing horticultural crops with high concentrations of these compounds [92,93]. However, the ascorbic acid concentration is not improved by blue or red light (Figure 4). Tocopherols, a type of vitamin E, are considered as potent antioxidants. Vitamin E, and most particularly α-tocopherol. Plays an essential role in protecting the photosynthetic apparatus from photo-oxidative stress and photoinhibition [94]. α-Tocopherol is the prevalent form of vitamin E and the most biologically active form for humans. It is a potent nutrient that prevents atherosclerosis, cancer, and central nervous system disorders [92]. α-Tocopherol levels fluctuate in response to environmental stress [95,96]. As an important environmental factor, light conditions influence α-tocopherol concentration in plants, that is, high light treatment improves α-tocopherol and γ-tocopherol content in *Arabidopsis* [97]. Moreover, additional blue light can enhance α-tocopherol content in beet microgreen and harvested tomato fruit and γ-tocopherol concentration in parsley microgreen [72,98]. In this study, blue light has a similar influence that α-tocopherol levels were significantly elevated in BLFR (Figure 4). Therefore, blue light can enhance tocopherol accumulation in tomato fruit. Besides, the β-tocopherol level was higher in BLFR and RDFR than in WHFR. *VTE4* is a member of the vitamin E-core pathway (Figure 11) and functions in shaping tocopherol composition [99,100]. Geranylgeranyl diphosphate reductase (GGDR) is also involved in vitamin E biosynthesis (MEP pathway), mediating the production of phytyl diphosphate (PDP) for vitamin E and chlorophyll biosynthesis [99,101]. The expression of enzyme genes in the vitamin E synthesis pathway, including these enzymes, was higher in BLFR or RDFR. Results suggested that blue light may increase α-tocopherol concentration and that blue or red light may increase β-tocopherol concentration, which are important for fruit quality, through the enhanced expression of genes in the vitamin E synthesis pathway in tomato fruit.

### 3.5. Effect on Ionome

All ionomes represent the mineral nutrient and trace element composition of an organism. Ionomics is a study of ionomes using high-throughput elemental analysis technologies [102]. Ionomic analysis of fruits is supposed to be valuable in evaluating fruit productivity and quality in tomatoes and other fruit crops [28,103,104,105]. The effects of environmental stress on ionome have been investigated in plants, including tomatoes [106,107,108,109,110]. There have so far been studies on the effects of light quality on some mineral elements in horticultural crops, including tomatoes [111,112], yet no reports about ionomic profile changes in response to different light qualities in tomatoes. Therefore, this study aimed to evaluate the influence of light quality on element accumulation and distribution in tomatoes using ionomics. To the authors’ best knowledge, because there are few reports on the effects of light quality on the elemental profile of plants, results could be important to offer fundamental information for not only tomato fruit but also plants.

First, PCA results of ionomic analysis indicated that light quality greatly affects ionomic profiling of tomato fruit, involving effects on a wide range of elements. There are elements on which the effect of light quality was similar and different between fruit and leaves (Figure 7), and some elements showed different fruit/leaf ratios among light qualities (Figure S2); therefore, the effects observed in the fruit indicate that the transport of ions from the soil or from other organs to the fruit is affected by light quality. Next, ionomics combined with genetics can identify genes that regulate the ionome [113]. This study classified differentially expressed genes based on functional annotation databases to focus on the genes associated with element transport and accumulation. Genes for protein family “Rieske [2Fe-2S] iron-sulfur domain” and “aromatic-ring-hydroxylating dioxygenase (2Fe-2S-binding site)” were enriched between BLFR and RDFR (Table 1). The two proteins have the molecular function of 2 iron, 2 sulfur cluster binding, and aromatic-ring-hydroxylating dioxygenase also has the function of iron ion binding. The expression of chlorophyllide a oxygenase (CAO) gene, responsible for chlorophyll b biosynthesis [114], was higher in BLFR (Table S1). Fe deficiency increases the expression level of *CAO* in apple seedlings [115]. Therefore, it can be speculated that the high *CAO* expression level in BLFR was induced by low iron concentrations in tomato fruit (Figure 7). The gene expression of lipoxygenase, an antioxidant enzyme that can be influenced by changes in plant oxidative status induced by iron stress [116,117,118], may also associate with lower Fe concentrations in BLFR. Iron deficiency leads to decreased activity of antioxidant enzymes, such as lipoxygenase [116,118,119]. In this study, lipoxygenase gene expression showed downregulation in BLFR (Table S2).

Differentially expressed genes between BLFR and WHFR were significantly enriched in the GO molecular function term of metal ion binding (Table 1), suggesting that genes of proteins capable of binding metal ions are influenced by light quality. Light quality affected the expression of many genes for metal ion-binding proteins with diverse functions (Table S2). These proteins have the function of binding with Fe, Mg, Ca, and Zn ions according to the GO. The result of ionomic analysis in tomato fruit indicated that the difference between BLFR and WHFR has little influence on their ion concentration; therefore, different light qualities may affect the availability of metal ions rather than the uptake and movement of ions.

**Figure 12:**
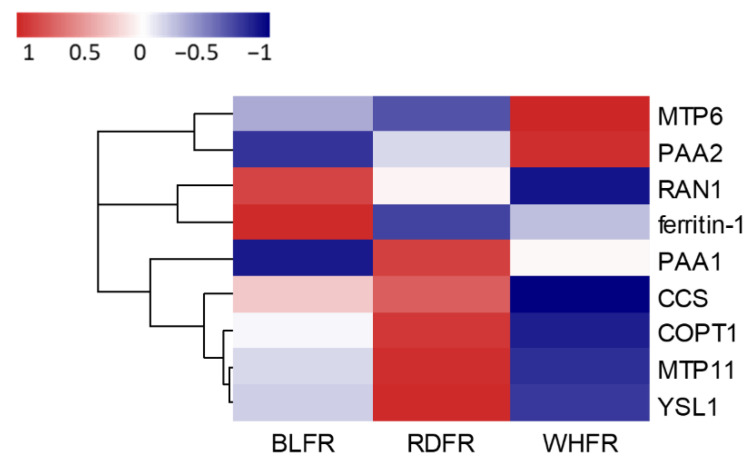
mRNA levels for genes involved in metal accumulation. Red and blue rectangles indicate higher and lower levels, respectively, than the mean of all treatments. Values indicate the z-score in the legend. Significant differences were found in the transcription level of *MTP6*, *MTP11*, *YSL1*, and *COPT1* in RDFR vs. WHFR, *ferritin-1* and *PAA1* in BLFR vs. RDFR, *PAA2* and *CCS* in WHFR vs. others, and *RAN1* in all comparison pairs. BLFR, RDFR, and WHFR refer to blue, red, and white light-treated fruits, respectively.

Ionomic analysis showed that Fe and Ni concentration in tomato fruit was high in RDFR. Some genes containing *MTP11* and *YSL1* with high expression in RDFR were found in the heatmap for metal tolerance proteins (MTPs) and metal transporters (Figure 12). The cation diffusion facilitator (CDF) family is a family of integral membrane divalent cation transporters involved in the transport of some ions containing Fe and Ni out of the cytoplasm either into the extracellular space or into internal compartments [120], and CDFs are also considered MTPs. Because MTP is upregulated in response to Fe treatment and to transport iron ions [121,122], *MTP11* may contribute to high Fe concentrations in tomato fruit in RDFR. Yellow stripe-like family (YSL) proteins have been identified due to the strong similarity of sequence with ZmYS1, which encodes a transporter in maize that functions in the root Fe-phytosiderophore uptake [123] and long-distance transport of ions [124,125]. YSLs play vital roles in iron homeostasis [126]. AtYSL1 plays a role in the long-distance transport of Fe via the xylem, and AtYSL2 transports iron and copper in the form of metal-nicotianamine (NA) [127]. YSLs also participate in Fe loading at diverse parts of plants, and YSL1 and YSL3 are proposed to translocate metals through vascular parenchyma cells in *Arabidopsis* [128,129]. The expression level of *YSL1* was 1.69-fold higher in RDFR than in BLFR (Figure 12), and Fe content in RDFR was significantly higher than in BLFR, suggesting that *YSL1* is possible to contribute to the high accumulation of Fe. In addition to Fe, YSLs also act as long-distance Ni-NA transporter in *Thlaspi caerulescens* [130]. Although the Ni concentration in leaves was lowest in RDFR, translocation of Ni to fruit improved in this treatment. Considering the role of YSL in translocation in various tissues [129] and the higher transcript level of *YSL1* (differentially expressed gene) in RDFR than WHFR, the high distribution and mobility of Ni to fruit may be attributed to the upregulation of *YSL1*. In contrast, ferritins are a class of proteins that can accommodate iron atoms and release them when needed for metabolic functions [131]. Overexpressed ferritin in plants can lead to enhanced iron concentration [132,133]. However, significantly elevated levels of *ferritin1* in BLFR (Figure 12) seemed to not increase the Fe content in tomato fruit. The expression of genes encoding vacuolar iron transporters (VITs), another metal transporter family, plays a significant role in iron transport and storage, supporting the transport of manganese in *Arabidopsis* [134]. However, the expression of their tomato homologs was not significantly different between light treatments, suggesting that VITs may not play a role in controlling Fe accumulation by light quality in tomato fruit (data not shown).

Cu accumulation presented a distinct pattern from Fe and Ni; that is, Cu concentrations in BLFR and RDFR were significantly lower than WHFR (Figure 7). Copper transporters (COPT), copper chaperones (CCH), and P-type ATPases are three members of functional categories of proteins that participate in Cu uptake and transport. COPT2, PAA1, PAA2, and CCH transcript levels in *Arabidopsis* were downregulated (to suppress Cu level) in response to ambient Cu excess and upregulated (to improve Cu level) in response to Cu deficiency to maintain Cu homeostasis in plants [135,136]. In contrast, Cu chaperones for superoxide dismutase (CCS) were upregulated in response to Cu excess [135,137]. In this study, transcripts of P-type ATPases (*PAA2*) were significantly downregulated in BLFR and RDFR compared to WHFR, and *CCS* showed lower transcript levels in BLFR and RDFR (Figure 12). The changes in these two genes indicated that they play the same roles with *AtPAA2* and *AtCCS*. In contrast, the expression level of SlCOPT1, which shows high similarity with AtCOPT2 [138], was significantly upregulated in RDFR than in WHFR. Although AtCOPT2 regulates Cu transport, the *COPT* transcript level is also inhibited by high Cu levels [136,139]. Therefore, low expression levels of *SlCOPT1* may result from high levels of Cu content in WHFR. According to the results, low Cu levels may be attributed to the cooperation of an expression change of *PAA2* and *CCS*. At the same time, it can be speculated that SlCOPT1 had little influence on Cu content in tomato fruit.

## 4. Materials and Methods

### 4.1. Plant Materials

Seeds of tomato (*S. lycopersicum* cv. Micro-Tom) were sown with a sterilized sumi-soil (Sumika Agrotech Co., Ltd., Osaka, Japan). Plants were grown under white fluorescent light for a 16 h photoperiod at 25 °C in a growth chamber until anthesis. The photosynthetic photon flux density (PPFD) of fluorescent light was 100 μmol m^−2^ s^−1^ while relative humidity and carbon dioxide concentration were not controlled. Plants were grown under blue LED light (peak at 470 nm) or red LED light (peak at 655 nm) in a growth chamber. White fluorescent light was used as a reference for LED treatments (Figure S1). LED treatments were performed using LED panels (ISLM, CCS, Inc., Kyoto, Japan), whereas a bulb-shaped fluorescent lamp (Neoball Z, Toshiba, Tokyo, Japan) was used as a white light treatment. The average PPFD on the planting pot surface was 100 μmol m^−2^ s^−1^ in LED treatments and 80 μmol m^−2^ s^−1^ in fluorescent light treatment. Plants were fertilized with 500 times diluted Hyponex stock solution (nitrogen: 6%, phosphoric acid: 10%, potassium: 5%, magnesium: 0.05%, manganese: 0.001%, boron: 0.005%, Hyponex Japan Corp., Ltd., Osaka, Japan) once weekly. The cultivation temperature was constant at 25 °C, and the photoperiod was 16 h. Red ripe fruits used for metabolomic and transcriptomic analyses were sampled 45 days after flowering (DAF), and whole fruit pericarps were frozen in liquid nitrogen and stored at −80 °C until use. Fruits used for ionomic analysis were sampled at 40 DAF, and leaves directly below the first flower cluster were sampled at 80 DAF. The samples were crushed after drying at 60 °C for 3 days for ionomic analysis. The cultivation was conducted with reference to Shibuya et al. [140].

### 4.2. Metabolomic Analysis

To analyze the metabolite fluctuations in whole fruit pericarp under different light treatments, metabolomic analysis was performed based on CE-TOFMS (Agilent CE-TOFMS system, Agilent Technologies, Santa Clara, CA, USA) analysis in cation and anion modes and LC-TOFMS (Agilent LC-TOFMS system, Agilent Technologies, Santa Clara, CA, USA) analysis in positive and negative modes were performed. Three biological replicates were prepared for each light treatment. For CE-TOFMS analysis, according to Ikeda et al. [23], 2000 mL methanol containing 200 μM of the internal standard was added to the sample prepared in *4.1* and crushed during cooling (1500 rpm, 120 s × 3). Crushed samples were mixed with 2000 μL chloroform and 800 μL Milli-Q water and centrifuged at 2300× *g* (4 °C, 5 min). Then, 200 μL supernatant was transferred into an ultrafiltration tube (Ultrafree MC PLHCC, HMT, centrifugal filter unit 5 kDa) and centrifuged at 9100× *g* (4 °C, 120 min). The filtrate was evaporated and dissolved in 50 μL Milli-Q water for measurement. For LC-TOFMS analysis, 1500 mL of 0.1% formic acid-acetonitrile containing 10 μM of the internal standard were added to the sample and crushed during cooling (1500 rpm, 120 s × 2). The samples were crushed again after adding 500 mL Milli-Q water (1500 rpm, 120 s) and centrifuged at 2300× *g* (4 °C, 5 min). The deposition was dissolved with 1500 μL of 1% formic acid-acetonitrile and 500 μL Milli-Q water and centrifuged, and the supernatant was mixed with the former supernatant. The supernatant was transferred to two ultrafiltration tubes (Nanosep 3K Omega, Pall), and ultrafiltration was performed by centrifuging at 9100× *g* (4 °C, 120 min). Phosphatide was removed using solid-phase extraction. After the filtrate was evaporated, it was dissolved with 200 μL of 50% (*v/v*) isopropanol water solution for measurement.

The peak obtained from CE-TOFMS and LC-TOFMS was automatically selected using MasterHands version 2.17.1.11 (Keio University, Tsuruoka, Japan). The detected peaks were searched in the HMT metabolite library and known-unknown library based on *m/z*, peak area, and migration or retention time. PCA, HCA, and heat map notation were performed using statistical analysis software developed by HMT.

### 4.3. Analysis of Total Phenolic Compounds

Extraction and assays were performed based on Anisworth and Gillespie [141]. Then, 100 mg sample and 1.9 mL ice-cold methanol were added to the microtubes and incubated in the dark at 25 °C for 24 h. Mixing was performed with a vortex mixer and incubated for another 24 h. After the samples were centrifuged at 13,000× *g* for 5 min, 100 μL supernatant was transferred to fresh microtubes. Gallic acid (standard) and 95% (*v*/*v*) methanol blank were added to other microtubes. Then, 200 μL of 10% (*v*/*v*) Folin-Ciocalteu reagent (20-fold dilution of Folin-Ciocalteu reagent from Wako Pure Chemical Corporation) were added to the tubes and vortexed thoroughly, and 800 μL of 700 mM Na_2_CO_3_ were added to each tube and incubated at 25 °C for 2 h. Then, a 200 μL sample, standard, or blank was transferred from the assay tubes to a clear 96-well microplate, and the absorbance of each well at 765 nm was determined using a microplate reader (SH-9000, Corona Electric, Hitachinaka, Japan).

### 4.4. Ionomic Analysis

Ionomic analysis was performed according to Shibuya et al. [28] and Sha et al. [142]. The whole pericarp of red-ripe tomato prepared in Section 4.1 was dried at 60 °C and ground to powder. The powder was digested with nitric acid and subjected to an elemental analysis with inductively coupled plasma-MS (ELAN DRC-e; Perkin Elmer, Waltham, MA, USA). PCA was performed using the function prcomp of R version 3.4.0 [143]. The comparison among treatments for the log_2_ ratio of leaves and fruits was tested by Tukey’s honestly significant difference in R version 3.4.0.

### 4.5. RNA-Seq

Total RNA was extracted from the whole pericarp samples using Cica Geneus RNA Prep kit (Kanto Chemical, Tokyo, Japan). The RNA-seq library was constructed with TruSeq Stranded mRNA LT Sample Prep Kit by Macrogen Japan Corp. The constructed library was sequenced on a NovaSeq 6000 S4 Reagent Kit. The raw reads were cleaned to produce trimmed data using Trimmomatic 0.38 [144]. GCF_000188115.4_SL3.0 was used as a reference genome to map cDNA fragments obtained from RNA-seq to the genome using HISAT2 version 2.1.0 [145,146,147]. The mapped reads were assembled using StringTie version 1.3.4d [145,148]. Differentially expressed gene analysis among nine samples was sorted into three pairs, BLFR vs. WHFR, RDFR vs. WHFR, and BLFR vs. RDFR. The significant results were selected based on conditions of an independent *t*-test raw *p* < 0.05. For significant lists with |FC| ≥ 2, HCA was performed to group similar samples and genes. GSEA was performed using the DAVID tool [149] based on GO, KEGG, and other functional annotation databases. Heatmap was performed using the package pheatmap [150] of R version 4.1.3 [143].

## 5. Conclusions

Hypothesizing that light quality during fruit development is a key factor influencing tomato fruit metabolism, this study found that metabolome, ionome, and transcriptome are greatly influenced by light quality. Amino acid metabolism, secondary metabolite biosynthesis, and Calvin cycle were active in blue light treatment. The concentration of Glu, at the central position of amino acid metabolism, was significantly increased by blue light in tomato fruit, promoting the accumulation of many other amino acids and related secondary metabolites. In addition, some antioxidants, such as phenols and tocopherols, were increased by blue light. Collectively, blue light affects the taste of fruit and nutritional value and improves tomato resistance to environmental stress by promoting the accumulation of various metabolites, and transcriptomic analysis indicated that a high concentration of these metabolites might be attributed to fluctuation in genes related to metabolite accumulation. Although the effect of red light has been mainly reported [18,19], this research is expected to contribute to the improvement of fruit quality by blue light. Element levels in fruits were more susceptible to changes in light quality than in leaves, and some trace elements were likely to be enriched in fruits under red light. The expression level of genes encoding metal ion-binding proteins, MTPs, and metal transporters is altered in response to different light qualities and therefore may regulate the accumulation of elements in tomatoes.

## Figures and Tables

**Table 1:** Enrichment analysis of Differentially expressed genes in three comparison pairs.

Comparison Pair	Category	Term	Count	*p*-Value
BLFR vs. RDFR	KEGG PATHWAY	sly01200: Carbon metabolism	8	0.00025
	KEGG PATHWAY	sly00710: Carbon fixation in photosynthetic organisms	5	0.00053
	KEGG PATHWAY	sly01110: Biosynthesis of secondary metabolites	11	0.03788
	KEGG PATHWAY	sly00260: Glycine, serine and threonine metabolism	3	0.04694
	GOTERM BP	GO:0009416~response to light stimulus	3	0.03245
	INTERPRO	IPR017941: Rieske [2Fe-2S] iron-sulfur domain	2	0.03144
BLFR vs. WHFR	GOTERM BP	GO:0006040 amino sugar metabolic process	3	0.00813
	GOTERM BP	GO:0005986 sucrose biosynthetic process	2	0.04521
	GOTERM BP	GO:0006000 fructose metabolic process	2	0.04521
	GOTERM MF	GO:0046872 metal ion binding	11	0.04589
	GOTERM MF	GO:0031409 pigment binding	3	0.02199
	KEGG PATHWAY	sly00710: Carbon fixation in photosynthetic organisms	6	0.00139
	KEGG PATHWAY	sly01200: Carbon metabolism	9	0.00769
	UP KEYWORDS	Carbohydrate metabolism	4	0.00658
RDFR vs. WHFR	UP KEYWORDS	Stress response	6	0.00009
	UP KEYWORDS	Fruit ripening	4	0.00101
	GOTERM BP	GO:0009835~fruit ripening	4	0.00098
	GOTERM BP	GO:0071470~cellular response to osmotic stress	2	0.03408
	GOTERM MF	GO:0005215~transporter activity	7	0.02876
	INTERPRO	IPR003854: Gibberellin-regulated protein	3	0.01839

Results from a GSEA. “Category” indicates original databases or resource where the terms orient. “Count” indicates number of genes in the gene set term. “*p*-Value” indicates modified Fisher’s exact *p*-value. BLFR, RDFR, and WHFR refer to blue, red, and white light-treated fruits, respectively.

## Data Availability

The data presented in this study are available on request from the corresponding author.

## Supplementary Materials

The following are available online at https://www.mdpi.com/article/10.3390/ijms232113288/s1.
