# Supplementary material for: Effect of Light Quality on Metabolomic, Ionomic, and Transcriptomic Profiles in Tomato Fruit
Source: Int J Mol Sci. 2022 Oct 31;23(21):13288. doi: 10.3390/ijms232113288 (PMC9654364; doi:10.3390/ijms232113288)
Supplement: Supplementary file 1 [file ijms-23-13288-s001.zip › ijms-1963206-supplementary.pdf]

Table S1. Differentially expressed genes involved in secondary metabolite biosynthesis between BLFR and RDFR.

		Comparative Analysis
Entrez Gene ID	Product	BLFR/RDFR
101259725	serine hydroxymethyltransferase 4	2.16*
104644439	dehydrodolichyl diphosphate synthase 2	2.06*
101265978	tropinone reductase homolog	-2.21*
100191110	glycerate dehydrogenase	2.52*
101250853	fructose-bisphosphate aldolase	2.61*
101249960	putative glucose-6-phosphate 1-epimerase	2.18*
101255353	very-long-chain enoyl-CoA reductase	3.16*
101261422	chlorophyllide a oxygenase, chloroplastic	2.26*
101055519	Hop-interacting protein THI032	2.13*
101244441	chlorophyllide a oxygenase, chloroplastic-like	2.46*
101260852	fructose-1,6-bisphosphatase 1, chloroplastic	2.42*

* indicates significant difference at $p < 0.05$ by an independent *t*-test .
A negative number in “Comparative Analysis” the value of RDFR/BLFR instead of BLFR/RDFR
BLFR and RDFR refer to blue and red light-treated fruits, respectively.

Table S2. Differentially expressed genes involved in metal ion binding between BLFR and WHFR.

Entrez_Gene_ID	Product	Comparative Analysis BLFR/RDFR		Comparative Analysis BLFR/WHFR		Comparative Analysis RDFR/WHFR	
101254639	probable pectate lyase 8	-1.54		2.25	*	3.47	*
101257228	peroxidase 51	-2.48		-2.07	*	1.20	
101256516	zinc finger protein ZAT5	-2.03		-3.70	*	-1.82	
100125906	2-oxoglutarate-dependent dioxygenase (ODD)	1.80	*	-6.61	*	-11.93	*
543994	lipoxygenase (LOX1.1)	-1.23	*	2.11	*	2.60	*
101257518	putative magnesium-protoporphyrin monomethyl ester cyclase	1.72	*	2.38	*	1.39	
101253681	gibberellin 2-beta-dioxygenase 8-like	1.19		-2.36	*	-2.82	*
543975	chlorophyll a/b-binding protein precursor (CAB4)	3.23	*	2.41	*	-1.34	
101243766	chlorophyll a-b binding protein 13, chloroplastic (CAB13)	2.88	*	2.46	*	-1.17	
101253648	peroxidase 42	1.00		-2.34	*	-2.35	*
101266527	chlorophyll a-b binding protein Cab9 (CAB9)	2.58		2.59	*	1.01	

* indicates significant difference at $p < 0.05$ by an independent *t*-test .
Negative numbers in “Comparative Analysis” indicate the value of RDFR/BLFR, WHFR/BLFR, and WHFR/RDFR, instead of BLFR/RDFR, BLFR/WHFR, and RDFR/WHFR, respectively.
BLFR, RDFR and WHFR refer to blue, red, and white light-treated fruits, respectively.

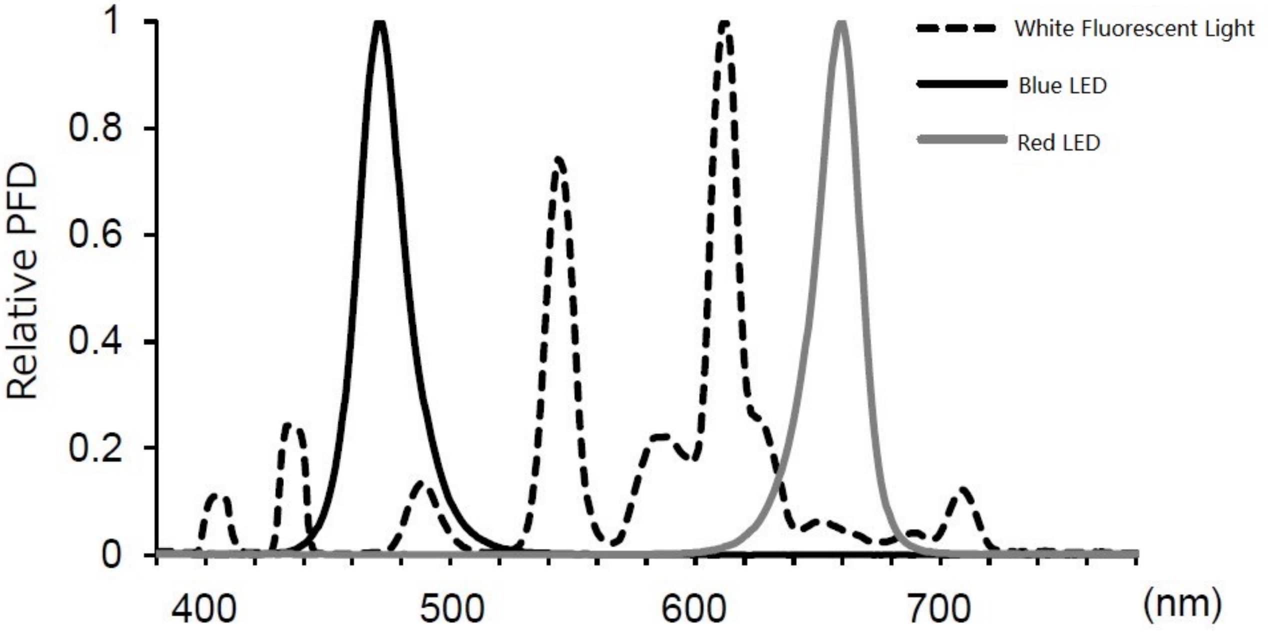

Figure S1. Fluorescent and LED light spectra.

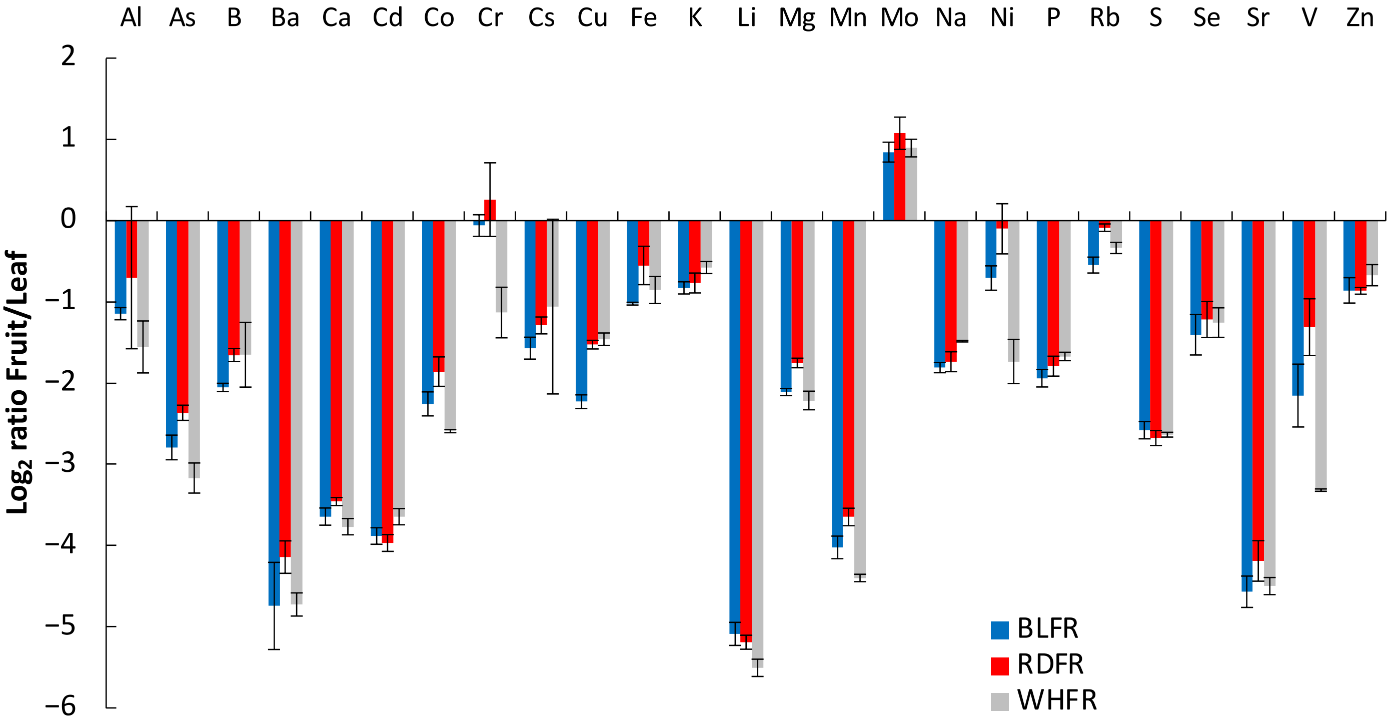

Figure S2. Ratio of elements in fruits vs. leaves. Results are means ± SE (n = 3). Different letters indicate significant differences at $p < 0.05$ by Tukey-Kramer's test. BLFR, RDFR, and WHFR refer to blue light treatment, red light treatment, and white light treatments, respectively.

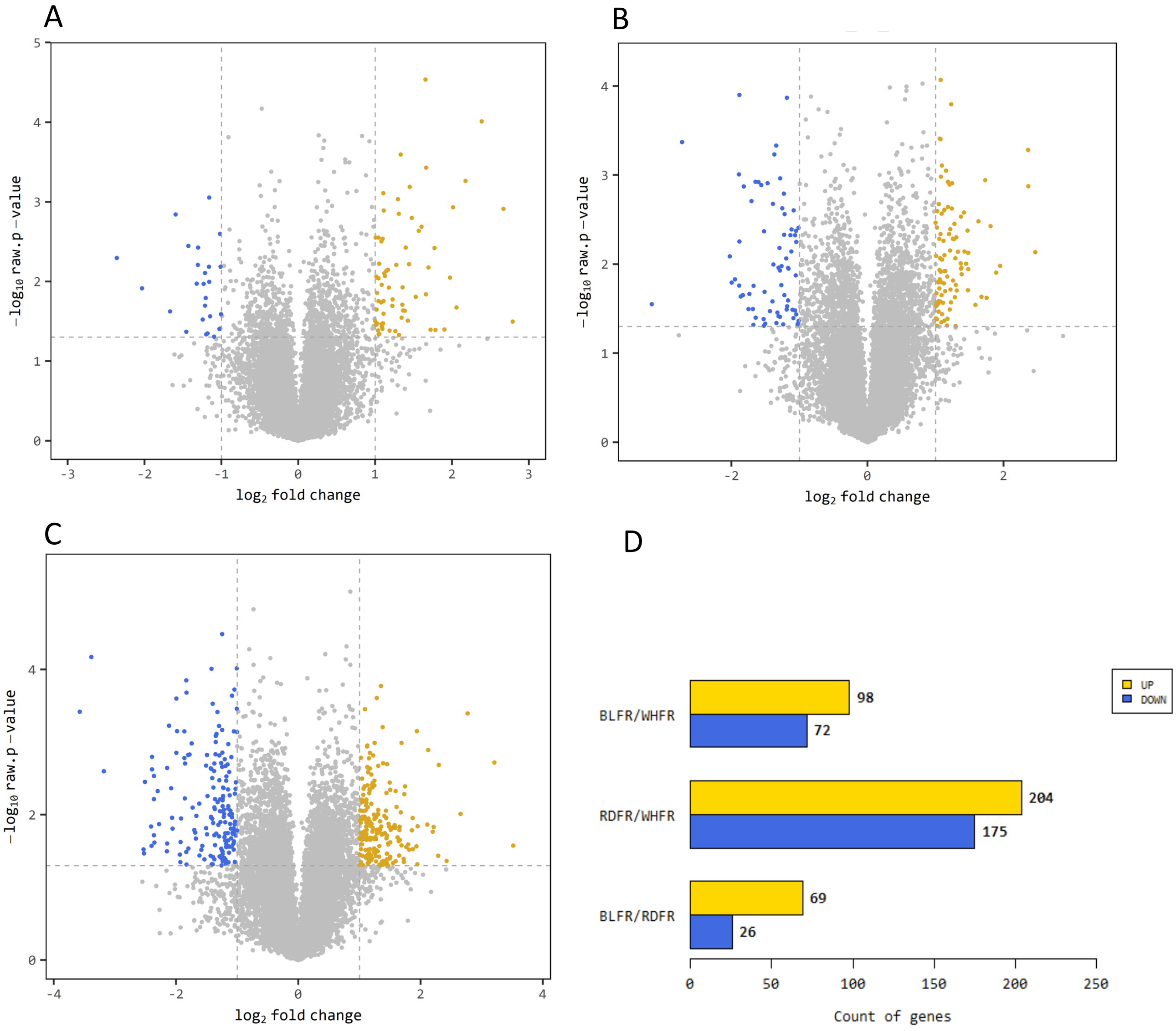

Figure S3. Differentially expressed genes are displayed in the volcano plot between BLFR and RDFR (A), BLFR and WHFR (B), RDFR and WHFR (C). Upregulated and downregulated genes (|FC|>=2 and $p < 0.05$) are displayed in the bar graph (D). BLFR, RDFR and WHFR refer to blue, red, and white light-treated fruits, respectively.
